# How pattern formation in ring networks of excitatory and inhibitory spiking neurons depends on the input current regime

**DOI:** 10.1186/1471-2202-14-S1-P123

**Published:** 2013-07-08

**Authors:** Birgit Kriener, Moritz Helias, Stefan Rotter, Markus Diesmann, Gaute T Einevoll

**Affiliations:** 1 Department of Mathematical Sciences and Technology, Norwegian University of Life Science, Ås, Norway; 2 Inst. of Neuroscience and Medicine (INM-6) and Inst. for Advanced Simulation (IAS-6), Jülich Research Centre and JARA, Jülich, Germany; 3 Medical Faculty, RWTH Aachen, Aachen, Germany; 4 Faculty of Biology, University of Freiburg, Freiburg, Germany; 5 Bernstein Center Freiburg, Freiburg, Germany

## 

Pattern formation, i.e., the generation of an inhomogeneous spatial activity distribution in a dynamical system with translation invariant structure, is a well-studied phenomenon in neuronal network dynamics, specifically in neural field models. These are population models to describe the spatiotemporal dynamics of large groups of neurons in terms of macroscopic variables such as population firing rates. Though neural field models are often deduced from and equipped with biophysically meaningful properties, a direct mapping to simulations of individual spiking neuron populations is rarely considered. Here, we consider networks with regular topologies, such as rings and lattices, where neuron positions are distributed on regular grids.

Neurons have a distinct identity defined by their action on their postsynaptic targets, i.e., they act either excitatorily or inhibitorily. When the distribution of neuron identities is assumed to be periodic, pattern formation can be observed, given the coupling strength is supercritical, i.e., larger than a critical weight.

Intriguingly, this critical weight is strongly dependent on the characteristics of the neuronal input, i.e., it depends on whether neurons are mean-driven or fluctuation-driven, and very different linearizations of the full non-linear system are relevant in order to assess stability.

We present and analyze these two linearizations, one that is derived directly from the leaky integrate-and-fire dynamics [1], the other from linear response theory in the diffusive coupling limit [2,3]. In the subcritical weak-coupling regime both approaches describe the firing rates of individual neurons with equally good precision, and by analysis of the respective linear stability we can predict under what conditions the system becomes unstable to spatial perturbations, and which spatial firing pattern will be assumed.

We moreover analyze the effect of structural randomness by rewiring individual synapses or redistributing weights.
